# Tuberculosis: An Update for the Clinician

**DOI:** 10.1111/resp.14887

**Published:** 2025-01-31

**Authors:** Saskia Janssen, Melissa Murphy, Caryn Upton, Brian Allwood, Andreas H. Diacon

**Affiliations:** ^1^ TASK Cape Town South Africa; ^2^ Radboud University Medical Center Nijmegen the Netherlands; ^3^ Tygerberg Hospital Cape Town South Africa; ^4^ Division of Pulmonology, Department of Medicine Stellenbosch University Cape Town South Africa

**Keywords:** diagnostics, post‐tuberculosis lung disease, prevention, treatment, tuberculosis

## Abstract

Tuberculosis (TB) remains a significant global health threat with high mortality and efforts to meet WHO End TB Strategy milestones are off‐track. It has become clear that TB is not a dichotomous infection with latent and active forms but presents along a disease spectrum. Subclinical TB plays a larger role in transmission than previously thought. Aerosol studies have shown that undiagnosed TB patients, even with paucibacillary disease, can be highly infectious and significantly contribute to TB spread. Encouraging clinical results have been seen with the M72/AS01_E_ vaccine. If preliminary results can be confirmed in ongoing larger trials, modelling shows the vaccine can positively impact the epidemic. TB preventive therapy (TPT), especially for high‐risk groups like people living with HIV and household contacts of drug‐resistant TB patients, has shown efficacy but implementation is resource intensive. Treatment options for infectious patients have grown rapidly. New shorter, all‐oral treatment regimens represent a breakthrough, but progress is threatened by rising resistance to bedaquiline. Many new chemical entities are entering clinical trials and raise hopes for all‐new regimens that could overcome rising resistance rates to conventional agents. More research is needed on the management of complex cases, such as central nervous system TB and severe HIV‐associated TB. Post‐TB lung disease (PTLD) is an under‐recognised but growing concern, affecting millions of survivors with lasting respiratory impairment and increased mortality. Continued investment in development of TB vaccines and therapeutics, treatment shortening, and management of TB sequelae is critical to combat this ongoing public health challenge.

## 1 Background/Epidemiology

Despite global efforts to control it, tuberculosis (TB) remains a significant threat to global health. TB remains the number one infectious disease killer globally, after being surpassed by coronavirus disease (COVID‐19) only briefly [1]. Every year more than 10 million new cases of TB, of which 0.5 million are drug resistant, are registered globally. Although TB is a curable disease, mortality remains high with 1.3 million deaths in 2022. TB is poverty‐related and disproportionally affects South‐East Asia and sub‐Saharan Africa, where it is the leading cause for the loss of disability adjusted life years (DALYs) [2]. We are off track to meet milestones set in the World Health Organisation (WHO) End TB Strategy to reduce TB incidence by 80%, TB deaths by 90%, and to eliminate catastrophic costs for TB‐affected households by 2030 [3]. Urgent action is required to improve case detection, TB prevention and treatment outcomes.

Historically, disease progression after infection with *Mycobacterium tuberculosis* (M.tb) were divided into latent TB, where individuals are asymptomatic and non‐infectious, and active TB, a symptomatic, potentially infectious disease, with only a minority of individuals clearing the infection without intervention. It is increasingly recognised that TB infection is a more nuanced spectrum of disease, ranging from non‐infectious M.tb infection, through subclinical TB (without symptoms, or without the individual being aware of or reporting their symptoms) which can be non‐infectious or infectious, to clinically active TB [4]. Based on TB prevalence surveys, approximately 50% of microbiologically confirmed TB cases have subclinical TB [5]. Combined with the increasing evidence of TB transmission, where subclinical TB appears to be as infectious as clinical TB [6], this is alarming and needs to be considered in TB detection algorithms. Current diagnostic algorithms fail to detect many patients transmitting M.tb. This is illustrated by aerosol sampling studies, where M.tb was isolated from individuals with incipient, undiagnosed TB at similar rates as patients with active disease [7]. The current focus on primarily screening people who cough (e.g., in contact investigations) should be reconsidered. Most aerosolization probably occurs during tidal breathing and cough does not increase the amount of M.tb released [8].

## 2 Diagnostics and Biomarkers

TB diagnosis typically begins with symptom screening, which is subjective and misses asymptomatic cases, and chest x‐ray, which requires infrastructure and trained personnel [9]. Recent advancements in this area include mobile low‐dose imaging and computer‐aided detection software [10]. Smear microscopy and culture were the standard for detection of M.tb for decades, but this has changed with the WHO endorsing molecular tests that is, nucleic acid amplification tests (NAATs) for detecting M.tb and rifampicin resistance, including Xpert MTB/RIF Ultra (Cepheid) and Truenat MTB (Truenat) [11]. Increasingly, diagnostic algorithms rely on these molecular tests, with the caveat that these tests do not distinguish between live and dead M.tb, and that disease due to non‐tuberculous mycobacteria (presenting with similar signs and symptoms as TB) may increasingly be missed. Due to the lower detection limit, Xpert MTB/RIF Ultra (Cepheid) has a higher sensitivity and is recommended particularly for paucibacillary forms of TB like central nervous system TB (CNS TB) [12]. Due to its paucibacillary nature, diagnosis of CNS TB still relies on a combined interpretation of clinical features, CSF chemistry and cytology, molecular tests like Xpert MTB/RIF Ultra and mycobacterial culture, paralleled with the exclusion of alternative diagnoses [13]. Diagnostic accuracy of TB diagnostic tests is improved with larger volumes of CSF analysed [14]. In addition, tests in development including Truenat MTB Plus (Molbio, Verna, India), SILVAMP TB LAM (FujiFilm, Tokyo, Japan) and metagenomic next‐generation sequencing show promising results [15, 16, 17].

For drug susceptibility testing (DST) of second line drugs, line probe assays (e.g., GenoType MTBDTplus) are widely used [18]. Culture‐based phenotypic DST remains the gold standard for extensive drug‐resistance detection, though molecular tests are available for broader drug resistance testing [19]. The Xpert MTB‐RIF assay is the most widely accessible molecular resistance test. Resistance to rifampicin (R) is strongly associated with multidrug‐resistant TB (MDR‐TB; resistant to isoniazid (H) and R). H monoresistance occurs three times more frequently than MDR‐TB, but undiagnosed H resistance favours development of MDR‐TB during continuation treatment with H and R [1, 20]. The newer automated moderate complexity NAATs, endorsed by the WHO, detect not only M.tb and R resistance but also H resistance—increased use of these tests will improve the identification of individuals with monoresistance [11]. Apart from this, a priority is to develop rapid, point‐of‐care tests for DST of the newer TB drugs including bedaquiline.

Where accessible, whole genome sequencing is increasingly being used for DST. Whole genome sequencing has important benefits to phenotypic DST, most importantly the shorter turn‐around time [21].

Most diagnostic tests are validated on sputum only. Alternative sample types, including tongue swabs, urine, stool, and aerosols, are being explored [22]. The WHO has endorsed the urine‐based AlereLAM test for TB diagnosis in PLWH with CD4 counts < 100 cells/μL [23].

There has been growing interest in measuring the host response to TB in recent years. Biomarker tests detecting blood RNA signatures and analytes in blood can distinguish between TB disease states [24, 25, 26], with RNA‐based biomarkers predicting disease onset up to 6 months in advance [27]. A point‐of‐care test (Xpert MTB Host Response) using fingerpick blood has also been developed but requires further investigation [28]. Recently, the WHO has also recommended using C‐reactive protein (> 5 mg/L) for TB screening in people living with HIV [29].

With the development of shorter regimens the need for well‐performing relapse prediction biomarkers is becoming increasingly clear. Where a high baseline mycobacterial load seems to be an important predictor of relapse [30, 31], prediction scores based on clinical information (body mass index, time to positivity of sputum cultures) and markers of host response seem to perform well [31, 32, 33, 34]. These scores require evaluation in larger prospective studies. An accessible, point‐of‐care relapse prediction score would accelerate the development of shorter and individualised TB treatment regimens.

## 3 Prevention

### 3.1 Vaccines

At present, the only licensed vaccine against TB is Bacillus Calmette–Guérin (BCG), which is typically administered to infants at birth and effectively protects against severe forms of TB [35, 36, 37]. However, its efficacy against pulmonary TB, the predominant form of the disease, is limited, with significant waning observed 10 years post‐infant vaccination [38]. Consequently, there is a need for a new TB vaccine with long‐lasting efficacy. At the time of the latest WHO Global TB report, 15 vaccine candidates were in clinical development, spanning a diverse array of platforms, including mRNA‐based vaccines entering phase 1 trials [1, 39]. Notably, some of these candidates are not only being evaluated for their efficacy in preventing TB disease or recurrence, but for their potential to prevent M.tb infection, as indicated by a positive interferon gamma release assay (IGRA) [40]. Among these candidates, the protein subunit vaccine M72/AS01_E_ shows promise. A phase 2b study demonstrated 49.7% efficacy (95% CI 2.1%–74.2%) in preventing progression to TB disease in HIV‐negative, IGRA‐positive adults [41]. A larger phase 3 multi‐country trial is now underway, including both IGRA‐positive and IGRA‐negative adolescents and adults, as well as an HIV‐positive cohort, with the completion of the trial expected in 2028. Although a 50% efficacy might seem modest, modelling studies suggest that such a vaccine could significantly impact TB control, particularly in the adolescent/adult population [42]. Despite absence of clear correlates of protection, recent studies in non‐human primates offer hope for a breakthrough [43, 44, 45]. It is also encouraging that most vaccine candidates in the pipeline are in phase 2b or phase 3 trials. Now, more than ever, the opportunity to achieve licensure for a new TB vaccine may be within reach. On the downside, our current approach to TB vaccine development will have to be critically reviewed and rebuilt if these vaccines fail, which will take many years. Key stakeholders have increased efforts to ensure continuity in TB vaccine development [46].

### 3.2 TB Preventive Therapy

**FIGURE 1:**
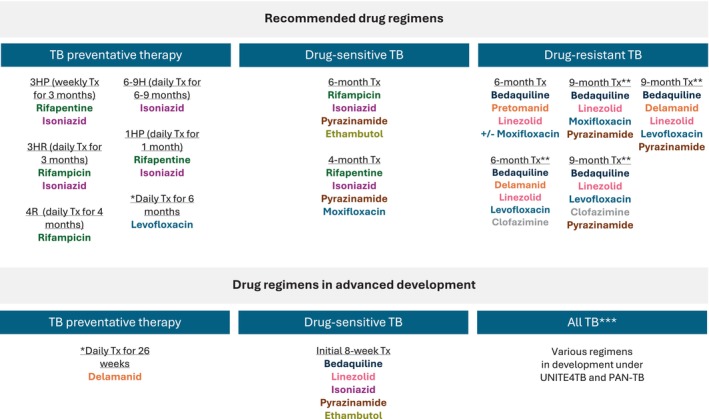
Current recommended regimens for TB prevention and treatment. This figure shows regimens that are currently recommended by the World Health Organisation, and in advanced development for tuberculosis (TB) preventive therapy, treatment of drug‐susceptible TB and drug‐resistant TB, respectively. Drugs are colour‐coded by their respective chemical class. *For drug resistant TB patient contacts; **Quinolone susceptibility required and *** Regimens in development within UNITE4TB and PAN‐TB contain novel compounds without cross‐resistance, that could be used as first‐line regimens in individuals with TB disease without prior knowledge of drug resistance profiles. H, isoniazid; P, rifapentine; R, rifampicin; Tx, treatment.

TB preventive therapy (TPT) significantly reduces the risk of progression from TB infection to active disease. TPT is recommended for all people living with HIV, where progress has been made in terms of coverage, but also for household contacts of TB patients (with emphasis on children under 5 years) and other risk groups including patients starting anti‐TNF treatment, organ or haematologic transplant recipients and dialysis patients [47]. Significant gaps need to be closed before United Nations (UN) targets are met [1]. Currently recommended regimens include a 3‐month regimen of once weekly rifapentine plus isoniazid (3HP), a 3‐month regimen of daily isoniazid plus rifampicin (3HR), or 6–9 months of daily isoniazid (6–9H) (Figure 1). A 1‐month regimen of daily rifapentine plus isoniazid (1HP) [48] or 4 months of daily rifampicin alone may also be offered as alternatives (4R) [47]. Whilst shorter rifapentine regimens 3HP and 1HP are safe and associated with high completion rates [48, 49, 50], adverse reactions are common during the first month of treatment. The most common reactions include flu‐like symptoms for 3HP and urticaria for 1HP; these are generally mild and self‐limiting [49, 50, 51, 52]. Pre‐treatment counselling about these reactions and their prognosis may further improve completion rates [50]. Hepatotoxicity occurs less with 3HP (0.4%) or 1HP (2% or less) compared to 9H [48, 49], but this remains a point of caution for all TPT regimens.

Two recent trials showed a reduction in incidence of TB with 6 months of daily levofloxacin after household exposure to drug resistant TB disease (MDR‐TB or RR‐TB, resistant to R and H or R only, respectively) [53, 54]. The TB‐CHAMP trial evaluated 6 months of levofloxacin to prevent MDR‐TB disease in children and adolescents, resulting in an incidence of MDR‐TB of 1.1% versus 2.6% in the placebo group, which was not statistically significant [54]. Similarly, the VQUIN trial, evaluating 6 months levofloxacin in adult individuals with MDR‐TB exposure, reported an incidence of TB disease in 0.6% in the levofloxacin group versus 1.1% in the placebo group [55]. Levofloxacin use was associated with an increased risk of musculoskeletal adverse events. In a meta‐analysis combining the findings of these trials, the difference in TB incidence did reach statistical significance, though owing to the low underlying TB rates the estimated number needed to treat to prevent one TB case remains high, particularly in adults [56]. Based on these two trials, 6 months levofloxacin is included in the updated WHO recommendations for TPT for RR‐TB contacts [47]. A Phase 3 trial evaluating the efficacy and safety of 26 weeks of daily delamanid in high‐risk household contacts of MDR TB patients is currently ongoing (PHOENIx MDR‐TB; NCT03568383). Delamanid TPT will hopefully address the gap of available regimens for contacts of patients with pre‐XDR and XDR‐TB with quinolone resistance.

Apart from TPT, nutritional support has also shown to be beneficial in reducing TB incidence in household contacts [57].

## 4 Treatment

### 4.1 Drug‐Susceptible TB

The recommended regimen for drug‐susceptible pulmonary and extrapulmonary TB was developed over 4 decades ago and consists of 6 months of rifampicin (R) and isoniazid (H) to which pyrazinamide (Z) and ethambutol (E) are added in the first 2 months. Although this regimen is safe and effective, the extended duration makes regimen completion challenging. A number of trials aiming to reduce treatment duration with fluoroquinolones failed to show non‐inferiority [58, 59, 60] until a 4‐month regimen including rifapentine, isoniazid, pyrazinamide and moxifloxacin (M) (4HPZM) was successful. This regimen is now WHO endorsed for treatment of DS‐TB in people aged 12 years and older [61, 62]. However, the persistent need for adherence support as well as the need for resistance testing to rifampicin and moxifloxacin limit potential gains in cost‐effectiveness [63], and accessibility of rifapentine remains limited.

Trials evaluating higher doses of rifampicin have so far failed to show increased efficacy of HRZE, although doses were mostly only minimally increased (up to 20 mg/kg), where the risk of drug‐induced liver injury may be increased with doses > 20 mg/kg [64].

### 4.2 Drug‐Resistant TB

In 2020 the Nix‐TB trial investigating BPaL, a novel 3‐drug regimen with bedaquiline, the nitroimidazole pretomanid and the repurposed antibiotic linezolid, was shown to cure patients with extensively resistant or difficult to treat DR‐TB in only 6 months, albeit in an uncontrolled study [65]. The TB‐PRACTECAL trial confirmed that BPaL with moxifloxacin (BPaLM) for 24 weeks was non‐inferior to the WHO standard of care for treatment of pulmonary MDR/RR‐TB and had a better safety profile [66, 67]. In 2022, WHO recommended BPaLM for 6 months as the new standard regimen for MDR/RR‐TB in patients aged 14 years or older without previous exposure to bedaquiline, pretomanid or linezolid. Importantly, excluding moxifloxacin from the regimen in patients with fluoroquinolone resistance does not require treatment extension [68].

BPaLM as a regimen free of injectable drugs and with a duration comparable to DS‐TB regimens was a breakthrough, though currently does not have safety and efficacy data in a subset of vulnerable patients. For children, adolescents, pregnant and breastfeeding women with MDR/RR‐TB, WHO recently endorsed a 6‐month regimen composed of bedaquiline, delamanid, linezolid, levofloxacin and clofazimine (BDLLfxC) tested in the BEAT‐TB study, demonstrating efficacy and safety in these groups. Levofloxacin or clofazimine can be excluded from the regimen according to fluoroquinolone resistance results [69, 70]. The EndTB study investigated combinations of bedaquiline, linezolid, levo‐ or moxifloxacin, delamanid, clofazimine and pyrazinamide [71]. Following early release of the successful EndTB study results, WHO provides guidance on three alternative injectable‐free 9‐month regimens for adults and children (BLMZ, BLLfxCZ, BDLLfxZ). However, these regimens require susceptibility to fluoroquinolones.

Patients not eligible for one of those regimens such as those with resistance or previous exposure to bedaquiline, pretomanid or linezolid of more than 1 month, or, for some regimens, fluoroquinolone resistance, still require individualised, much longer treatments with poorer efficacy and tolerability.

### 4.3 TB Therapeutics in Development

That shorter regimens are possible using a risk‐stratified treatment strategy was elegantly shown in the TRUNCATE trial [30]. Selected and carefully monitored patients with mild disease and without risk factors for failure or recurrence received an 8‐week regimen with first and second‐line drugs (bedaquiline, linezolid, isoniazid, pyrazinamide and ethambutol) that was noninferior to standard treatment. This confirmed that even with conventional agents most patients can be cured in considerably less than 24 weeks. How to identify this minority of ‘hard‐to‐treat’ individuals that drive the longer treatment duration in current regimens is the subject of ongoing research.

**FIGURE 2:**
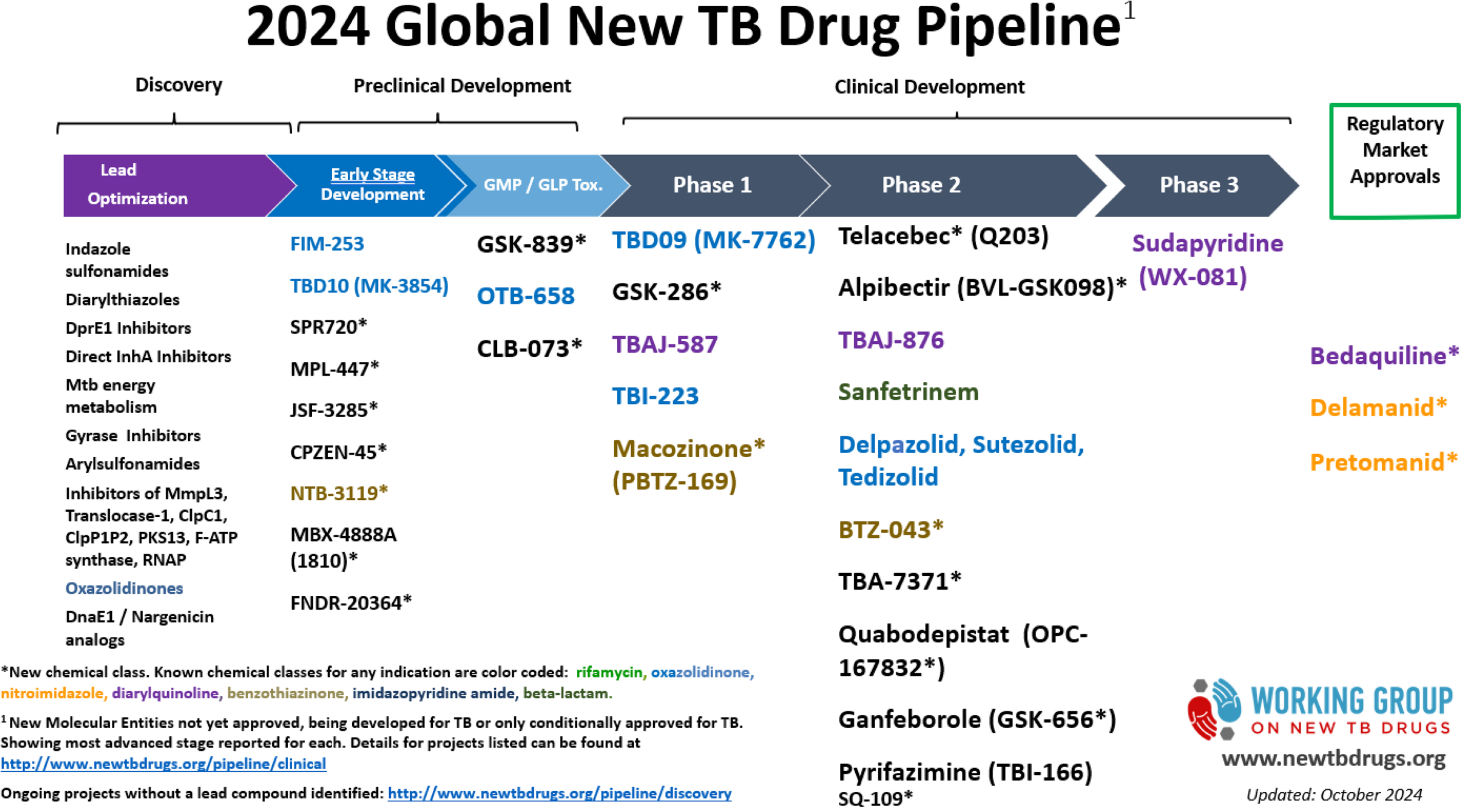
Global TB drug pipeline. This figure shows an overview of new therapeutic compounds for TB at different stages of development. From Stop TB Partnership's Working Group on New Drugs [72].

Large international initiatives such as UNITE4TB (https://www.unite4tb.org) and PAN‐TB (https://www.pan‐tb.org), which comprise private, academic, and industry partners, are taking advantage of the extensive list of novel drugs to develop shorter regimens for all patients (Figure 2 [72]). In accordance with recently released WHO product target profiles these regimens exclude current first‐line agents and aim for broad suitability, and are designed to meet the efficacy, safety, and tolerability needs of the majority of TB patients while remaining cost‐effective and accessible for national TB programs [73].

**FIGURE 3:**
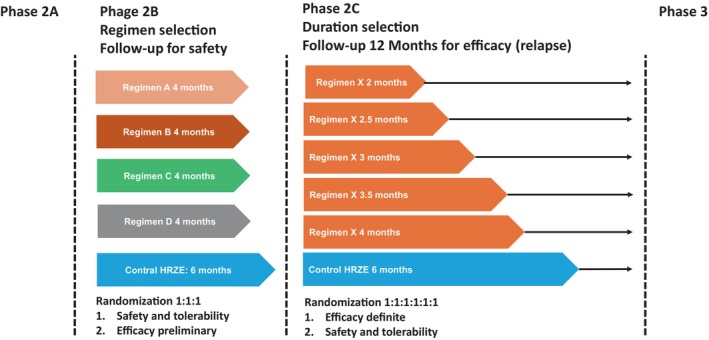
Trial schedule for accelerated development of TB treatment regimens. This figure shows a trial schedule illustrating the design of accelerated development of TB treatment regimens in the PAN‐TB and UNITE4TB projects. Regimens that contain combinations of new agents first undergo a 4‐month Phase 2B study to ascertain safety and preliminary efficacy. The best performing regimen enters a duration‐randomisation Phase 2C with clinically relevant endpoints to evaluate further treatment shortening potential based on relapse rates, enabling selection of the best candidates and treatment durations for a Phase 3. Adapted from Hoelscher et al. [76].

Conceivably, it is impossible to test all permutations of drug combinations at different dose levels and durations in clinical trials. A consensus is emerging whereby suitable combinations are selected based on in vitro testing, murine experiments and extrapolation of drug concentrations expected at site of disease in different metabolic conditions [74, 75]. Prioritised regimens would first undergo a 4‐month Phase 2B study to ascertain safety of the novel combination and obtain an early reading of potential efficacy. The best performing regimens would enter a duration‐randomisation phase with clinically relevant endpoints to evaluate further treatment shortening potential based on relapse rates. This would produce the best candidates and treatment durations for a phase 3 study leading to adoption of a novel regimen as shown in Figure 3 [74, 76].

Phase 2C studies with duration randomisation also offer a unique opportunity to identify risk factors for treatment failure and relapse, that is, profiles for ‘harder‐to‐treat’ patients. Such biomarkers could pave the way to a stratified medicine approach with individual treatment durations that cure each patient with a high probability instead of one treatment duration that has a high probability of curing most patients. This could increase cost‐effectiveness dramatically even if some patients still needed treatment beyond 4 months [77].

A hopefully minor setback are rapidly rising resistance rates in the field against bedaquiline [78, 79]. First published in 2004 the drug had a novel mechanism of action targeting mycobacterial energy metabolism. It was the first drug developed in decades in response to increasing resistance against first‐ and second line agents that could no longer be contained by public health measures [80, 81]. Bedaquiline received marketing registration in 2012 [82, 83], but was adopted into clinical guidelines only slowly and used mostly as a salvage agent, as such often partnered with weak or failing other drugs [78, 79, 84]. Alarmingly, bedaquiline is a cornerstone of the current standard regimen for RR‐TB and of all exploratory new regimens. Some hope comes from the alternative diarylquinoline molecules TBAJ‐587 and TBAJ‐876 that might overcome the most prevalent types of bedaquiline resistance (associated with mutations in the Rv0678 gene [85]).

### 4.4 Treatment of Extrapulmonary TB

To date, there are no separate treatment guidelines for extrapulmonary TB nor are the various disease manifestations included in trials with novel regimens [61]. Standard regimens will suffice for paucibacillary forms such as lymph node and pleural TB. Manifestations with increased morbidity and mortality risk are CNS‐TB, disseminated severe HIV‐associated TB, TB pericarditis and bone and joint TB. CNS‐TB is associated with a high mortality (around 50% for HIV‐infected patients) and long‐term neurologic sequalae in many survivors [86, 87, 88]. Most international guidelines recommend treating drug‐susceptible CNS‐TB with the same regimen as pulmonary TB for a duration of 9–12 months [61, 89]. Although high‐quality reviews have been published [90], the latest international guidelines specifically for CNS‐TB were published in 2009 [91]. New guidelines may suggest using an optimised dosage of rifampicin of > 20 mg/kg based on pharmacokinetic and safety findings from phase 2 trials [92, 93, 94]. For paediatric patients, a recent systematic review reported higher treatment success of a 6 month regimen including isoniazid and rifampicin, both at higher doses, pyrazinamide and ethionamide compared to the standard 12 month regimen (2HRZE/10HR) [95]. This regimen is now included in WHO guidelines. There are no clinical trials in patients with drug resistant CNS TB; these patients still need to be treated with longer regimens of 18–20 months where drugs need to be selected for their CNS penetration [96]. While adjunctive corticosteroids reduce mortality in adults with TB meningitis without HIV infection [91] a recent large clinical trial did not find a reduction in mortality in HIV‐patients with TB meningitis [86].

Another subgroup with high mortality are patients hospitalised with HIV‐associated TB [97]. In these patients, mycobacteremia is common and associated with a sepsis‐like syndrome with increased coagulopathy and immune activation [97, 98, 99, 100]. The ongoing NEW‐STRAT TB trial will inform whether adjunctive steroids, or increased dosage of rifampicin and addition of levofloxacin during the first 2 weeks of treatment will improve survival [101].

### 4.5 Management of Paradoxical Reactions

Paradoxical reactions to M.tb during effective pharmacological treatment can occur in individuals with and without HIV infection. In patients without HIV infection corticosteroids are preferred, and some literature exists on the use of alternative agents including anakinra [102], infliximab [103, 104] or thalidomide [104, 105], however more evidence is needed prior to recommending their use. HIV‐associated paradoxical TB immune reconstitution inflammatory syndrome (IRIS) can be safely managed with prednisolone at a dosage of 1.5 mg/kg/day for 2 weeks followed by 2 weeks of 0.75 mg/kg/day [106]. Paradoxical TB IRIS can be prevented with a preventive course of prednisolone when starting antiretroviral therapy in individuals with CD4 counts < 100 cells per microliter [107].

## 5 Post TB Lung Disease

It is often not appreciated that the impact of TB does not end at treatment completion, and post‐TB complications will profoundly impact on the wellbeing of many survivors. There were an estimated 155 million TB survivors alive in 2020, or 1 in 50 people [108], with 10 million people added to this number annually [1]. Up to 60% will have measurable respiratory impairment [109] with possibly severe economic, social and psychological consequences [110]. TB‐survivors have an approximately three fold increase in mortality compared with age matched peers [111], and almost half of the total DALYs associated with TB will occur after ‘successful’ treatment completion, with an estimated 5.8 DALYs per person in the post‐TB period alone [112]. Patients surviving TB have a four‐fold risk of lung cancer [109] and may experience acute exacerbations of their lung disease, analogous to exacerbations in other chronic lung diseases (e.g., COPD) [113].

Post‐TB lung disease (PTLD), the best recognised consequence of TB, is currently defined as ‘evidence of chronic respiratory abnormality, with or without symptoms, attributable at least in part to previous TB’ [114]. This broad definition allows for both the identification of early (pre‐symptomatic) disease, and the co‐existence of PTLD with other chronic lung disease (e.g., smoking related COPD), however is hampered by a lack of specificity. PTLD is unfortunately a poorly understood, heterogeneous disease, with marked variation both between individuals, but also between different areas of the lung within an individual. The current clinical patterns described include parenchymal disease (fibrosis, destruction, cavitation); airway disease (bronchiectasis and small airway disease); pleural disease; as well as post‐TB pulmonary hypertension. It is not yet clear what impact these different clinical patterns have on outcomes, and thus, they cannot yet be designated as phenotypes [114]. Exacerbations of PTLD have not yet been defined, but currently should be considered to include increased dyspnoea, sputum production and sputum purulence, for which the patient seeks medical attention [115]. The causes of PTLD exacerbations are poorly understood. Importantly, PTLD exacerbations can be difficult to distinguish from TB recurrence, which occurs with increased frequency in TB survivors [116]. Chronic pulmonary aspergillosis, although beyond the scope of this review, is an important complication of PTLD and can be indistinguishable from PTLD itself [117]. Thus, a high index of suspicion is needed for both testing and initiation of anti‐fungal therapy (if available).

A recent scoping review highlighted the lack of evidence for interventions to either prevent or treat PTLD [118]. Thus, current management guidelines for PTLD cannot be considered evidence based. Expert opinion recommends vaccination and pulmonary rehabilitation as likely beneficial, with management as per bronchiectasis and COPD guidelines for large and small airway PTLD, respectively. Inhaled corticosteroids should be avoided because of risk of TB reactivation or disease due to non‐tuberculous mycobacteria [119].

## 6 Conclusion

TB continues to have a major impact on global health and disproportionately affects low‐ and middle‐income countries. Accumulating evidence suggests that subclinical TB is an important driver of transmission and future research should inform on the optimal management of subclinical TB. In the field of diagnostics, development of accessible DST for the newer drugs is a priority. More research is also needed to develop an effective vaccine and expand options for TPT for contacts of patients with DR‐TB. After decades of stagnation, the development of a 4 month regimen for DS‐TB and all‐oral, 6–9 month regimens for DR‐TB mark major advancements. The fact that all DR‐TB regimens include bedaquiline, paired with the increasing resistance to the drug is alarming and underlines the need for ongoing investment in research and development of new TB therapeutics, with diagnostics in parallel. Other research gaps are the optimal management of high‐risk groups with extrapulmonary TB including patients with CNS‐TB and severe HIV‐associated TB. PTLD has been added to the research agenda only recently despite its large burden, for which we still lack improved diagnostic criteria, preventive as well as therapeutic interventions. Continued investments are needed to fill these gaps and make a world free of TB a more realistic scenario, bringing us closer to targets set by the WHO End TB Strategy [3].

## Author Contributions

**Saskia Janssen:** conceptualization (equal), writing – original draft (lead). **Melissa Murphy:** writing – original draft (equal), writing – review and editing (equal). **Caryn Upton:** writing – review and editing (equal). **Brian Allwood:** writing – original draft (equal), writing – review and editing (supporting). **Andreas H. Diacon:** conceptualization (equal), supervision (equal), writing – original draft (equal), writing – review and editing (lead).

## Conflicts of Interest

The authors declare no conflicts of interest.
